# The Hippo signaling effector WWTR1 is a metastatic biomarker of gastric cardia adenocarcinoma

**DOI:** 10.1186/s12935-019-0796-z

**Published:** 2019-03-29

**Authors:** Jing Wei, Lincui Wang, Jun Zhu, Aiqin Sun, Guanzhen Yu, Miao Chen, Pan Huang, Hanqing Liu, Genbao Shao, Wannian Yang, Qiong Lin

**Affiliations:** 1 School of Medicine, Jiangsu University, Zhenjiang, Jiangsu China; 2 Changzheng Hospital, Shanghai, China; 3 Department of Pathology, Zhenjiang First People’s Hospital, Zhenjiang, Jiangsu China; 4 School of Pharmacology, Jiangsu University, Zhenjiang, Jiangsu China

**Keywords:** WWTR1, Hippo signaling, Gastric cardia adenocarcinoma, Metastasis, Prognostic biomarker

## Abstract

**Background:**

Gastric cardia adenocarcinoma (GCA) is an aggressive subtype of gastric cancer with a high metastatic rate. However, the metastatic biomarker of GCA has not been established.

**Methods:**

To search for the biomarker for GCA metastasis, we here examined expression of the Hippo signaling effector WWTR1 (WW domain containing transcription regulator 1, commonly listed as TAZ) in tumor tissue samples from 214 GCA cases using the tissue microarray assay (TMA), and statistically analyzed association of the WWTR1 expression with metastasis-related pathological outcomes and cumulative survival of the GCA patients. Furthermore, shRNA knockdown was used to determine the role of WWTR1 in promoting cell migration in gastric cancer cells.

**Results:**

The results have shown that WWTR1 is overexpressed in 66.4% of the GCA tumor samples. Expression of WWTR1 has a significant inverse correlation with cumulative survival of GCA patients (p < 0.01). WWTR1 positive patients had a mean survival of 56.9 ± 4.4 months, comparing to WWTR1 negative mean survival of 77.3 ± 5.9 months. More importantly, expression of WWTR1 significantly associated with tumor invasion and metastasis (in T stage, p = 0.031; N stage, p < 0.01; and TNM stage, p < 0.001). Furthermore, knockdown of WWTR1 impaired migration of gastric cancer AGS cells.

**Conclusions:**

Our studies have identified WWTR1 as a metastatic biomarker of GCA for poor prognosis, defined a role of WWTR1 in driving metastasis of gastric cancer, and suggested WWTR1 as a potential target for anti-metastatic therapy of GCA.

## Background

Gastric cardia adenocarcinoma (GCA) belongs to the type II gastroesophageal junction adenocarcinoma (GEJAC), the most aggressive type of gastric carcinoma [1–3]. Surgery is still the best treatment for GCA, no effective chemo or targeted therapy is currently available. However, the post-surgery recurrence rate of the TNM stages I/II GCA patients is as high as 46% [4]. GCA patients with distant metastasis have a very low survival rate, about 2–12% in the 5-year survival rate [1]. Clinical data indicate that metastasis is the major cause for poor prognosis and low survival rate of GCA [1, 4, 5]. Thus, targeting metastasis is pivotal for developing new therapeutic strategies in GCA treatment to improve the survival rate of GCA patients.

Biomarkers associated with GCA prognosis or progression have been investigated. Down-regulation of expression of some genes, such as *RASSF2*, *RASSF6*, *FBXO32*, *GADD45A*, and *GADD45G*, by methylation has been found to associate with poor prognosis of GCA [6–9]. Expression of some known proto-oncogenic proteins, such as c-MET, HER2, PIM-3, MYC, SIRT1, and CHOP proteins, has been observed in correlation with progression of GCA [10–15]. In addition, specific microRNAs or long non-coding RNAs (lnc-RNAs) have been observed involved in progression of GCA [16–18]. Despite a number of biomarkers associated with poor prognosis or progression are established, few of metastatic biomarkers, especially metastatic driver proteins, in GCA have been identified.

We recently found that expression of the E3 ubiquitin ligase NEDD4 (also named as NEDD4-1) and the Hippo-YAP/WWTR1 (commonly listed as TAZ) signaling target gene product CYR61 (also named as CCN1) is significantly associated with metastasis of GCA and correlated with poor survival of GCA patients [19, 20]. Both NEDD4 and CYR61 functionally promote gastric cancer cell migration and invasion, suggesting that both NEDD4 and CYR61 are driver proteins for metastasis [19, 20]. Interestingly, NEDD4 has been found to ubiquitinate and down-regulate the Hippo signaling kinase LATS1, thus activate the YAP/WWTR1 mediated transcription [21]. These studies suggest that the Hippo-YAP/WWTR1 signaling may play a major role in promoting metastasis in GCA. Further investigation of the Hippo signaling in GCA metastasis is necessary to forward our understanding of the mechanism underlying GCA progression, improve diagnosis, prognosis and therapy of GCA, and develop anti-metastatic drugs for GCA targeted therapy.

WWTR1 is a downstream effector of the Hippo signaling and a transcriptional co-activator of the transcriptional factor TEAD [22–24]. Overexpression of WWTR1 has been observed in multiple types of solid tumors, including breast cancer, lung cancer, gastric cancer, colon cancer, renal cancer, liver cancer, ovarian cancer, pancreatic cancer, prostate cancer, melanoma, glioma, and sarcoma [25, 26]. Numerous studies have shown that WWTR1 promotes tumor initiation, growth, invasion and metastasis in breast and lung cancers [27–30]. WWTR1 is involved in multiple cancer cell signaling pathways, including WNT/CTNN, Ga12/Ga13/Rho, mevalonate/geranylgeranylation, mTOR, and fluid shear signaling pathways, that regulate cell proliferation, EMT, angiogenesis, drug resistance of tumors [31–37]. Our previous studies have shown that WWTR1 has a role in breast cancer cell migration and invasion [33], and demonstrated that CYR61, a WWTR1 target gene product, is a prognostic biomarker of GCA, and expression of CYR61 is associated with metastasis of GCA [20]. These studies strongly suggest that WWTR1 might be a valid biomarker and a potential therapeutic target for metastasis of GCA.

In this report, we examined expression of WWTR1 in tumor samples from 214 GCA cases using tissue microarray assay (TMA) and statistically analyzed association of WWTR1 expression with cumulative survival of GCA patients and clinicopathological data. We found that WWTR1 expression is positively correlated with tumor invasion and metastasis of GCA and inversely associated with cumulative survival of GCA patients. Knockdown of WWTR1 by expression of the *WWTR1* shRNA in gastric cancer AGS cells significantly inhibited cell migration, suggesting that WWTR1 might be a metastatic driver in GCA. Our studies have established WWTR1 as a biomarker for prediction of poor prognosis and a potential molecular target for anti-metastatic therapy of GCA.

## Results

### WWTR1 is highly expressed in GCA tumors and the expression is inversely correlated with cumulative survival

**Fig. 1:**
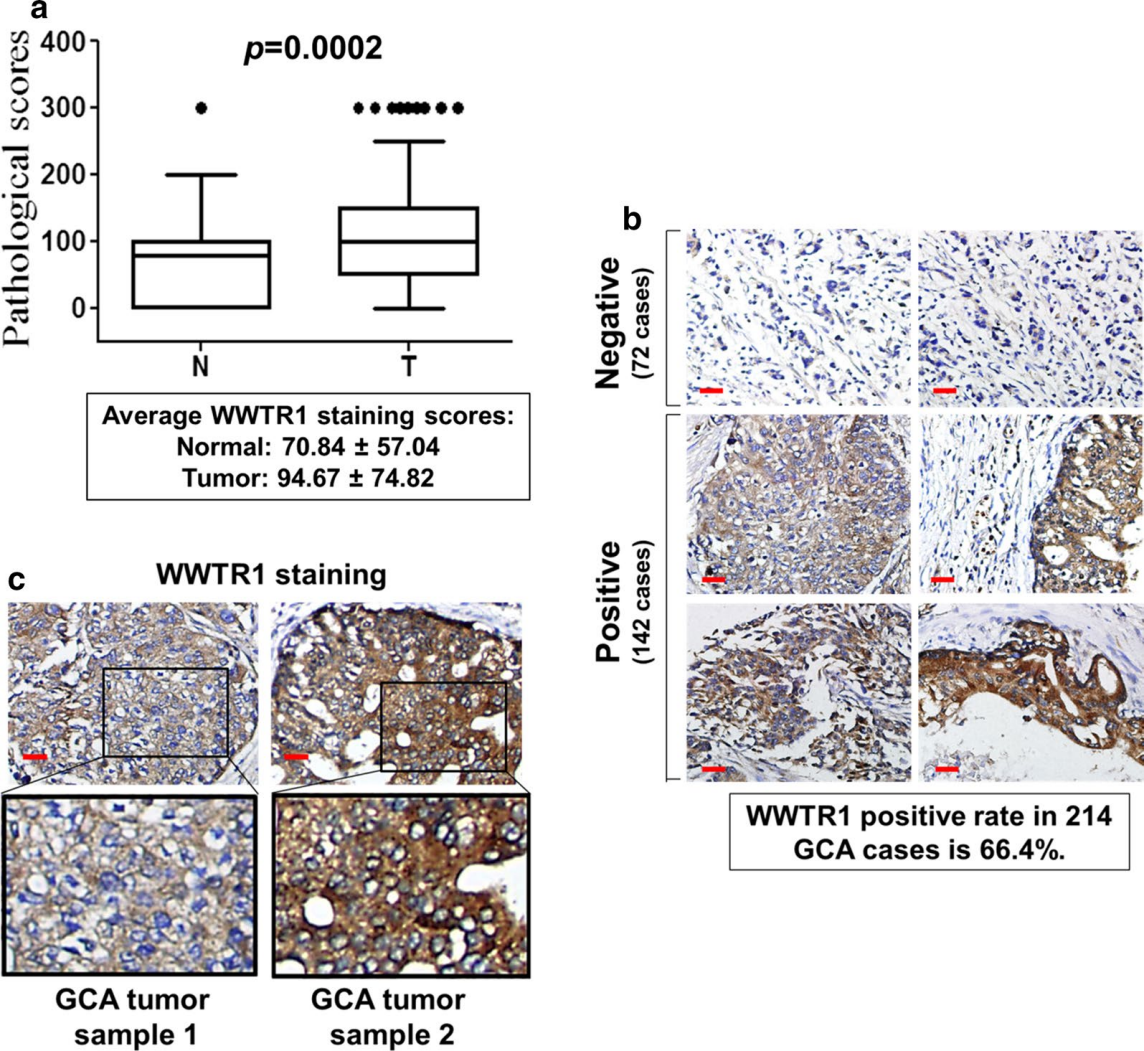
WWTR1 is overexpressed in GCA. **a** Expression of WWTR1 in GCA tumor tissue is significantly higher than in their adjacent normal gastric cardia tissue. The score distribution of IHC staining of WWTR1 in both GCA tumors (T) and their adjacent normal tissue (N) is shown in the box plots. The black dots at top are scores outside of the box plots. The mean scores and standard deviation are shown at bottom of the box plots. **b** IHC staining of WWTR1 in GCA tumor samples. Both WWTR1 negative (the top two panels) and positive (the bottom four panels) tumor samples are shown. Bar, 50 μm. **c** Two differentially stained WWTR1-positive GCA tumor samples with enlarged panels show no specific nuclear localization of WWTR1 in the tumor cells. Bar, 40 μm

Expression of WWTR1 in both the GCA tumor tissues and the adjacent normal tissues from 214 GCA cases was detected by IHC staining using tissue microarray assay (TMA) (Fig. 1). As shown in Fig. 1a, the average IHC staining score of WWTR1 in the GCA tumor tissue is 94.67 ± 74.82 while in the normal tissue is 70.84 ± 57.04 (*p* = 0.0002), indicating that expression of WWTR1 is significantly higher in the tumor tissue than in the normal tissue.

The expression ratio of WWTR1 in GCA tumors was further determined. The TMA assay showed that tumor samples from142 cases in total of 214 GCA cases (66.4%) were positively stained with anti-WWTR1 as shown in the bottom 4 panels in Fig. 1b, and 72 cases (33.6%) had little or no staining as shown in the top two panels in Fig. 1b, indicating that WWTR1 is overexpressed in GCA with a high frequency. In addition, staining of WWTR1 in GCA tumor cells was distributed all over the cell, no specified cellular localization was seen (Fig. 1c).

**Fig. 2:**
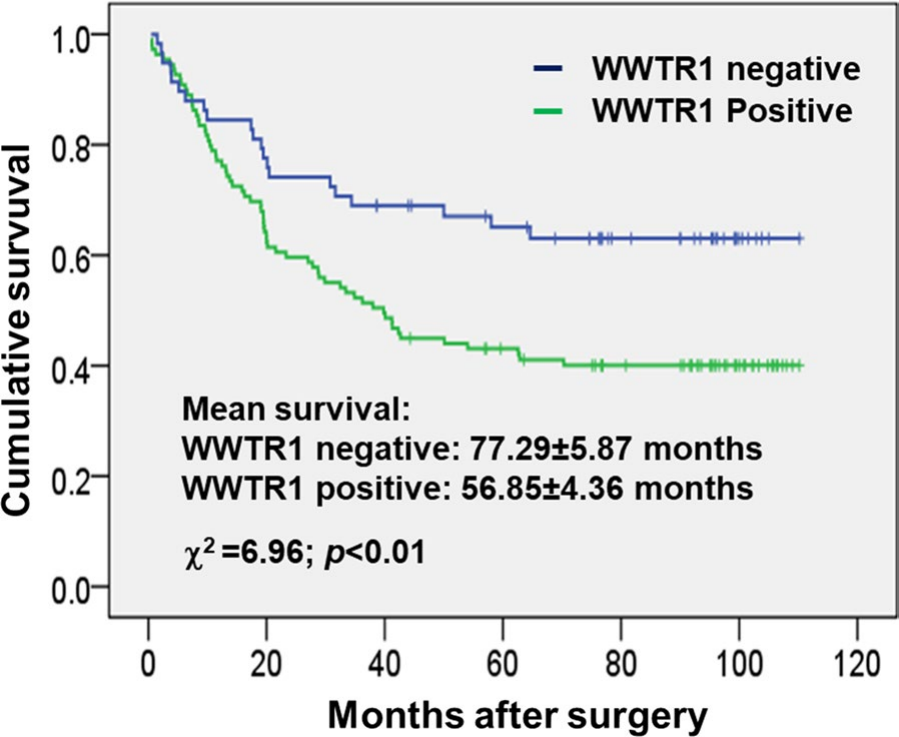
Overexpression of WWTR1 is reversely correlated with cumulative survival of GCA patients. Kaplan–Meier survival curve of the WWTR1 positive and negative GCA patients. The Chi square and *p* values from the Mantel-Cox test and the mean survival for the WWTR1 negative and positive GCA are shown in the figure. The *p*-value was calculated from Mantel-Cox test

To assess the role of WWTR1 in prognosis, we determined the association of WWTR1 expression with cumulative survival of the post-surgery GCA patients by statistical analysis. As shown in the Kaplan–Meier survival graph (Fig. 2), the patients with the WWTR1 negative tumor had average cumulative survival of 77.29 ± 5.87 months observed in the follow-up. On the other hand, the WWTR1 positive patients had an average cumulative survival of 56.85 ± 4.36 months (Fig. 2). Difference between the WWTR1 positive and negative patient’s cumulative survival was determined by the log-rank test (Fig. 2). The Chi square value is 6.96 with *p *< 0.01, indicating that cumulative survival between the WWTR1 positive and the negative patients is dramatically different and that WWTR1 expression is significantly inversely associated with post-surgery survival of GCA.

### Expression of WWTR1 is associated with tumor invasion and metastasis of GCA

We further statistically analyzed correlation of WWTR1 expression in clinicopathological characteristics in GCA tumors. As shown in Table 1, expression of WWTR1 is significantly associated with T stages (tumor invasion) (*p *= 0.031), N stages (lymph node metastasis) (*p *< 0.01), and TNM stages (combined with T, N, and the remote metastasis M) (*p *< 0.001), however, insignificantly associated with gender (p = 0.70), tumor size (*p *= 0.552), and grade (differentiation) (*p *= 0.503). The WWTR1 positive staining has been detected in 70.9% of T3/T4 patients, 72.3% of the N1–3 stages patients, 81.8% of the TNM stage IV patients and 73.5% of TNM stage III patients, compared to that in 55.6% of the T1/T2 patients, 54.8% of the N0 stage patients, 50% of the TNM stage I patients and 53.8% TNM stage II patients (Table 1). These clinicopathological data strongly suggest an association of WWTR1 expression with tumor invasion and metastasis in GCA, particularly with remote metastasis as WWTR1 expressed in TNM stage IV tumors with a significantly high frequency (81.8%). Furthermore, high expression of WWTR1 seems to occur more frequently in older GCA patients (> 60 years) than in younger patients (≤ 60 years) (p = 0.054) (Table 1).

**Table 1:** Association of WWTR1 expression with clinicopathological categories of GCA tumors

Clinicopathological category	Case number	WWTR1 positive	%	*p*
Age				
≤ 60	108	65	60.2	*0.054*
> 60	106	77	72.6	
Gender				
Male	157	103	65.6	0.7
Female	57	39	68.4	
Tumor size (cm)				
≤ 6	166	110	66.3	0.552
> 6	48	32	66.7	
T stage				
T1/T2	63	35	55.6	*0.031*
T3/T4	151	107	70.9	
N stage				
N0	73	40	54.8	*< 0.01*
N1–3	141	102	72.3	
Differentiation				
Well/Mod	130	84	64.6	0.503
Poor/Undiff	84	58	69.0	
TNM stage				
I	44	22	50.0	I/II to III/IV *< 0.001*
II	39	21	53.8	
III	98	72	73.5	
IV	33	27	81.8	

### WWTR1 is an important driver for gastric cancer cell migration

**Fig. 3:**
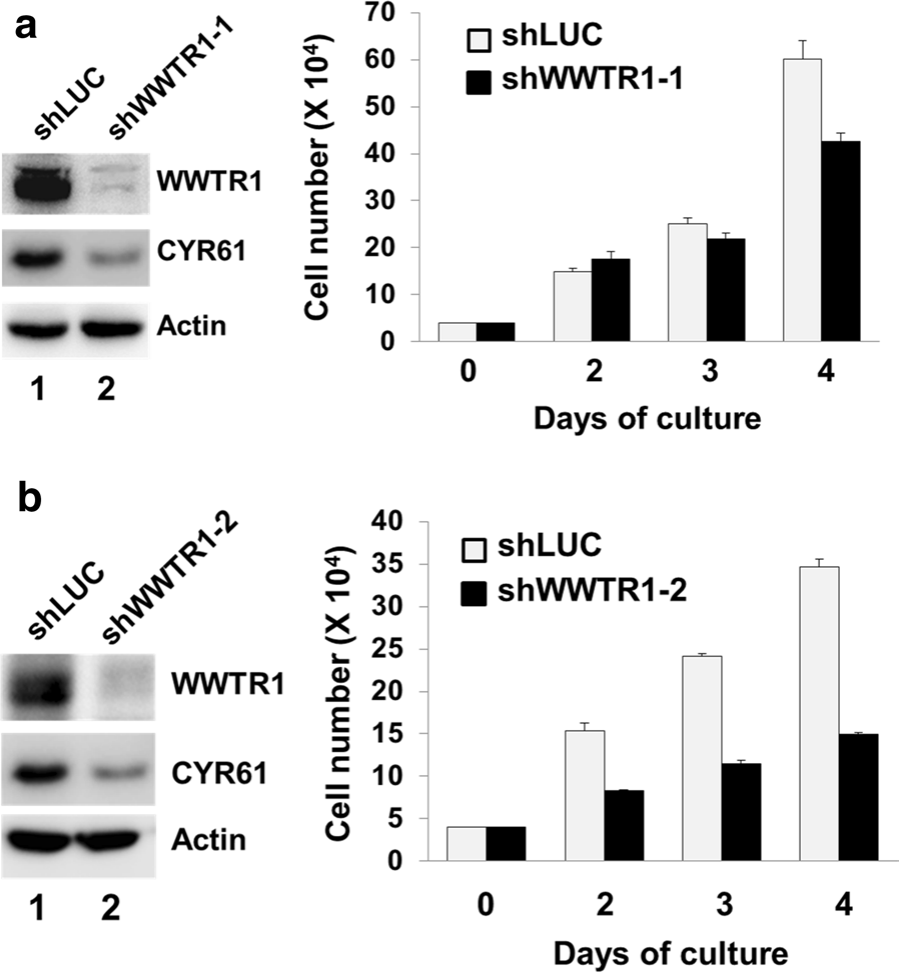
Knockdown of WWTR1 partially inhibits proliferation of AGS cells. Endogenous WWTR1 in AGS cells was depleted by lentiviral vector-loaded shWWTR1-1 or shWWTR1-2 for 48 h and detected by immunoblotting with anti-WWTR1 and anti-CYR61 from the cell lysates. The effect of WWTR1 knockdown on cell proliferation was quantified by counting the cell number under a phase microscope with a hemocytometer. The data used for quantification were from three independent experiments. **a** The effect of shWWTR1-1 on knockdown of WWTR1 and AGS cell proliferation; **b** the effect of shWWTR1-2 on knockdown of WWTR1 and AGS cell proliferation

To verify the role of WWTR1 in GCA, we set to determine if WWTR1 is directly driving GCA cell in proliferation and migration, a major cellular process in metastasis. We used AGS, which is a highly metastatic gastric cancer cell line [38], to mimic GCA cells in determination of the effect of WWTR1 knockdown in cell proliferation and migration. We first established two WWTR1-knockdown cell lines in AGS using lentiviral vector-loaded WWTR1 shRNAs. More than 90% of WWTR1 was depleted in both shWWTR1 cell lines (Fig. 3). Depletion of WWTR1 down-regulated expression of its target gene product CYR61 (Fig. 3a, b), indicating that the transcriptional activator function of WWTR1 is inhibited. Knockdown of WWTR1 caused a partial inhibitory effect (30–60% of the control cells) on cell proliferation after 4 days culture (Fig. 3a, b), suggesting that WWTR1 plays a partial role in AGS cell proliferation.

**Fig. 4:**
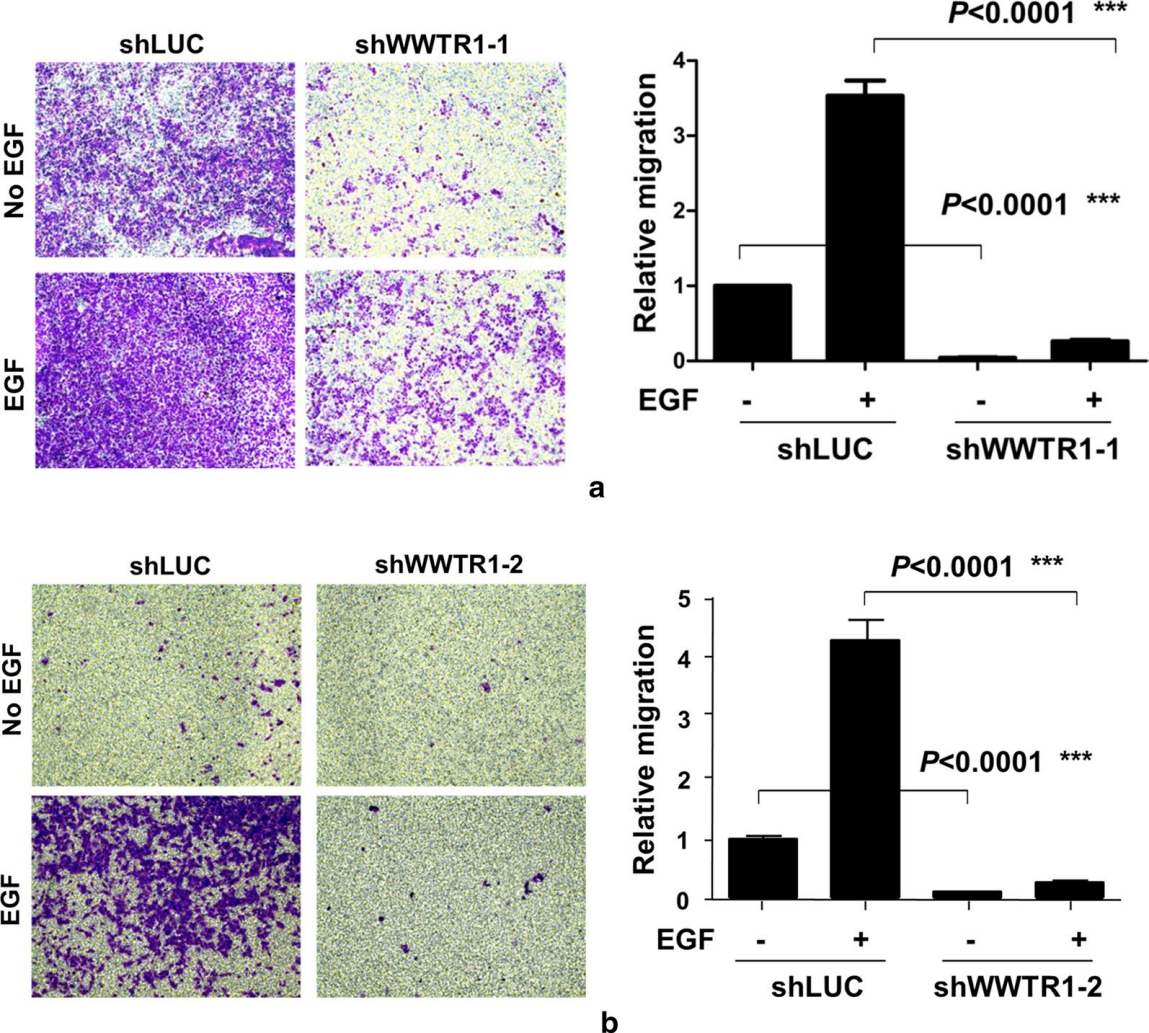
WWTR1 is required for gastric cancer AGS cell migration. The transwell assay is used to determine the effect of WWTR1 knockdown on AGS cell migration. The gastric cancer cell migration assays were repeated three times. For quantification of the migration rate in the transwell assay, the migrated cells (bottom side of the membrane) were fixed and stained by the crystal violet. The cells were counted under a microscope from three randomly selected fields. **a** The effect of shWWTR1-1; **b** the effect of shWWTR1-2. EGF (50 ng/ml) was used for stimulation of cell migration. ***p < 0.001

Next, we determined the effect of WWTR1 knockdown on cell migration in both shWWTR1 cell lines using the transwell migration assays. As shown in Fig. 4a, b, treatment of the control shRNA cell line with EGF induced a marked increase in cell migration, suggesting that EGF receptor (EGFR) signaling might be involved in gastric cancer metastasis. Upon knockdown of WWTR1, the basal and the EGF-promoted cell migration in both shWWTR1 cell lines were significantly inhibited, indicating that WWTR1 plays a pivotal driving role in AGS cell migration.

Taken together, the results from the IHC staining of GCA tumor tissues and the gastric cancer cell migration assay of the WWTR1-knockdown AGS cells suggest that WWTR1 is not only a clinical predictive index protein for poor prognosis of GCA but also a driver protein in metastasis of GCA.

## Discussion

Establishment of prognosis biomarker for the post-surgery GCA patients and identification of therapeutic target molecules and pathways are very important and urgent for improvement of survival rate of GCA, particularly of advanced stage GCA. Our previous studies have initially identified the WWTR1 target gene product CYR61 as a prognosis biomarker and an associated factor of metastasis of GCA, and a driver protein for gastric cancer cell migration [20]. In this report, we have shown that the WWTR1 is highly expressed in 66.4% of tumors from total of 214 GCA cases by IHC staining using the TMA assay. Expression of WWTR1 is significantly associated with poor prognosis and reversely correlated with cumulative survival of the post-surgery GCA patients. Analysis of clinicopathological characteristics shows that WWTR1 expression is significantly associated with GCA tumor invasion (T stage), lymph node metastasis (N stage), and remote metastasis (TNM stage IV). Knockdown of WWTR1 in the gastric cancer AGS cells resulted in severe impairment in both the basal and the EGF-stimulated cell migration. The results in this report have suggested a role of WWTR1 in driving metastasis of GCA and established WWTR1 as a predictive biomarker for poor prognosis of GCA.

WWTR1 is overexpressed in multiple types of solid tumors and its expression is associated with metastasis of breast cancer, lung cancer and melanoma [25, 26]. The mechanism underlying WWTR1-promoted tumor metastasis has been investigated in varies types of cancer cell lines and with a metastatic mouse model [39, 40]. Currently, there are several signaling pathways have been proposed involved in WWTR1-promoted tumor metastasis. It has been observed that WWTR1 activates EMT signaling pathway that promotes tumor growth, migration, and invasion by co-activating TEAD-mediated EMT gene transcription [34]. WWTR1 also mediates the RhoGTPase signaling that modulates cytoskeleton and promotes cell migration and invasion [32]. It is noticed that WWTR1 enhances cancer cell drug-resistance that may sustain cancer cell survival in bloodstream and increase the cancer cell deposition in the remote metastatic tissue [39]. Nevertheless, all these effects of WWTR1 are dependent on its co-transcriptional activity with TEAD [41]. However, the exact downstream target that mediates the WWTR1-promoted metastasis in GCA currently remains inconclusive. Based on our previous studies showing that CYR61 has very similar patterns in association with GCA metastasis and cumulative survival [20], we propose that CYR61 is a major target gene product mediating the WWTR1-promoted metastatic effect in GCA. CYR61 might transduce the metastatic effect by modulating cell adhesion receptors such as integrins that control cell migration and cell surface receptors such as VEGFRs that stimulate angiogenesis. We will follow the hypothesis and further determine the mechanism by which the WWTR1/CYR61 signaling axis promotes cancer cell migration, invasion and metastasis in future studies.

The upstream signaling that regulates WWTR1 may play a key role in promoting WWTR1-mediated metastatic effect [39]. Currently, it is not clear whether the Hippo kinase cascade or the non-Hippo signaling pathway regulates the WWTR1 transcriptional activity in GCA or gastric cancer AGS cells. It has been identified that the G-protein coupled receptor signaling, mainly through activation of Gα12/Gα13/RhoA, regulates the Hippo pathway to activate YAP/WWTR1 [32]. Our previous studies have shown that geranylgeranylation signaling suppresses the Hippo kinase cascade and activates YAP/WWTR1 in the estrogen receptor (ER)-negative breast cancer cells [33]. In addition, the other downstream effector of the Hippo signaling YAP, similar to WWTR1, also promotes gastric cancer cell migration and metastasis [42–44]. The transcriptional profile study indicates that both YAP and WWTR1 activates transcription of the same set of genes involved in cell migration, invasion and division, including *CYR61* [45]. These studies suggest that the Hippo signaling might be a major signaling pathway that controls the YAP/WWTR1-involved cell migration and invasion in gastric cancer.

Furthermore, we found that EGF stimulated AGS cell migration and knockdown of WWTR1 impairs both EGF-stimulated and EGF-independent AGS cell migration (Fig. 4), suggesting that WWTR1 might mediate both EGFR-dependent and –independent gastric cancer cell migration. A recent study has shown that some gastric cancer cell lines including AGS are resistant or partially resistant to treatment of cetuximab, an inhibitory EGFR antibody for treatment of metastatic colorectal cancer, on EGF-stimulated cell migration and invasion [46]. The WWTR1-activated cell migration signaling might mediate the cetuximab-resistance in these gastric cancer cell lines. It is interesting to examine the role of WWTR1 in cetuximab-resistant cell migration and invasion in these gastric cancer cell lines in our future studies.

The data in this report has established WWTR1 as a prognosis biomarker of GCA. More importantly, our studies here also provide a potential target for anti-metastatic therapy of GCA. Inhibition of the WWTR1 activation or its transcriptional co-activator activity, or interruption of interaction of WWTR1 with TEAD might be an effective approach for reducing and preventing metastasis and relapse of GCA after surgery, thus improving survival rate of GCA patients. Our previous studies have shown that geranylgeranylation plays a key role in activation of the YAP/WWTR1 transcriptional co-activator activity in breast cancer cells and migration and invasion of gastric cancer cells [20, 33]. Thus, the HMG-CoA reductase inhibitors such as statins or the geranylgeranyl-transferase inhibitors (GGTIs) that block geranylgeranylation could be used for treatment or prevention of metastasis of GCA. In addition, inhibitors that interrupt the interaction of WWTR1 with TEAD might also be effective to impede metastasis of GCA and could be used for the anti-metastatic therapy of GCA. It is inspiring to pursue these anti-metastatic therapeutic approaches in future clinical trials for GCA patients.

## Conclusions

Our studies have shown that WWTR1 is overexpressed in gastric cardia adenocarcinoma, expression of WWTR1 is reversely correlated with cumulative survival of GCA patients and significantly associated with GCA tumor invasion and metastasis. Furthermore, knockdown of WWTR1 markedly inhibits migration of gastric cancer AGS cells, suggesting a driving role of WWTR1 in metastasis. Thus, WWTR1 is a metastatic biomarker of GCA and its expression may be used for prognosis in clinic.

## Materials and methods

### Materials

Anti-TAZ (4883S) was purchased from Cell Signaling; anti-CYR61 (SC-13100) from Santa Cruz; anti-actin (RLM3028) from Ruiying Biological. The WWTR1 and luciferase (control) shRNA oligos were synthesized by ShengGong Company. IHC staining S-P kit (KIT-9710) was purchased from MAIXIN Biology Corporation. Atorvastatin calcium was purchased from WuXi Sigma. Transwell dishes were purchased from Corning Inc. Matrigel was purchased from BD Biosciences. The gastric cancer cell line AGS was purchased from the American Type Culture Collection (ATCC).

### The GCA tissue microarrays and the human GCA tissue specimens

The GCA tissue microarrays were made from 214 cases of the human GCA curative resection tissue specimens from the Gastric Cancer Tissue Bank at Department of Oncology, Changzheng Hospital (Shanghai, China). Collection of the tissue specimens was processed with patient informed consent, and the use of the GCA specimens and the associated clinicopathological information was approved by the Changzheng and Changhai Hospital Institutional Review Board. Clinicopathological information of the 214 GCA tumor samples has been shown in our previous report [19] (also shown in Table 1). For the GCA tissue microarray, two to three 2 mm-cores of the tumor tissue were selected to represent the status of the whole section. Adjacent normal tissue samples were also collected.

Among the GCA tumor specimens, about three quarters were from male patients; more than three quarters were equal to or smaller than 6 cm; about two-thirds were highly invaded (T3/T4) or metastasized to lymph nodes (N1–3) or at advanced TNM stages (III/IV). The post-surgery cumulative survival of the GCA patients was followed, censored, and described in previous studies [19]. The overall mean survival time of the patients is 64.036 ± 3.554 months with a 95% confidential interval (CI) ranged from 57.070 to 71.002 months. Tumor sizes (≤ 6 cm vs > 6 cm), invasive degrees (T1/T2 vs T3/T4), lymph node metastasis (N0 vs N1–3), differentiation grades (well/moderate vs poor/undifferentiated) and TNM stages (TNM I/II vs TNM III/IV) are all significantly and inversely associated with patient cumulative survival (*p *= 0.003 or < 0.001). The Chi square values from the log-rank test indicate that the TNM-stage (χ^2^ = 50.396) is the most significant one inversely associated with cumulative survival, followed by N stage (χ^2^ = 38.832) and T stage (χ^2^ = 27.661), suggesting that metastasis is the major factor associated with mortality of the GCA patients.

### Immunohistochemistry (IHC) staining

The IHC staining was performed as following: sections (4 μm in thickness) of paraffin-embedded GCA tissue microarrays were de-paraffinized and rehydrated in xylene and alcohol bath solution. The slides were pretreated with 0.01 M citrate buffer (pH 6.0) at 98 °C for 5 min using a microwave oven for antigen unmasking, then cooled to room temperature. To eliminate endogenous peroxidase the slides were incubated in 3% hydrogen peroxide for 10 min, then washed in 10 mM PBS (pH 7.4). The slides were blocked with normal goat serum at room temperature for 10 min, then with the anti-WWTR1 antibody (dilution: 1:100) at 4 °C overnight. Staining of the slides was performed using an IHC staining S-P kit (KIT-9710; MAIXIN Biology Corporation, Fuzhou, China) followed by counterstaining with hematoxylin. Expression of WWTR1 in the specimens was evaluated by two individuals scoring the staining under an Olympus CX31 microscope (Olympus, Center Valley, PA). The method for evaluation of the IHC staining was the same as previously described [19].

### Knockdown of WWTR1 by the shRNA

HEK293T cells were cultured and maintained in DMEM (Hyclone) supplemented with 10% FBS at 37 °C, 5% CO_2_. The gastric cancer AGS cells were grown in F12 K (Boster bio) supplemented with 10% fetal bovine serum (Excell bio), 100 U/ml penicillin, and 100 mg/ml streptomycin in 5% CO2 at 37 °C. Based on two targeting sequences in *WWTR1* cDNA (5′-G C C C T T T C T A A C C T G G C T G T A-3′) and (5′-G C G A T G A A T C A G C C T C T G A A T-3′), we synthesized two WWTR1 shRNA oligos: shWWTR1-1 (Forward: 5′-C C G G G C C C T T T C T A A C C T G G C T G T A C T C G A G T C A G C C A G G T T A G A A A G G G C T T T T T G-3′; Reverse: 5′-A A T T C A A A A A G C C C T T T C T A A C C T G G C T G T A C T C G A G T A C A G C C A G G T T A G A A A G G G C-3′) and shWWTR1-2 (Forward: 5′-C C G G G C G A T G A A T C A G C C T C T G A A T C T C G A G A T T C A G A G G C T G A T T C A T C G C T T T T T G-3′ Reverse: 5′-A A T T C A A A A A G C G A T G A A T C A G C C T C T G A A T C T C G A G A T T C A G A G G C T G A T T C A T C G C-3′) and cloned them into the lentiviral shRNA expression vector pLKO.1-TRC. The oligos were inserted into the AgeI/EcoRI sites of the vector.

For lentiviral particle packaging, HEK293T cells (1 × 10^6^) were seeded in a 35 mm tissue culture dish overnight. The lentiviral shRNA plasmid (pLKO.1-shWWTR1) was co-transfected with psPAX2 (Addgene) and pMD2.G (Addgene) into HEK293T cells for 8 h. The medium was harvested every day after transfection for 3 days, centrifuged at 1250 rpm for 5 min to remove cell debris, and used for infecting the AGS cells.

For infection, AGS cells (1 × 10^5^) were seeded in a 35 mm tissue culture dish overnight, infected with 1 ml of lentiviral particle medium in presence of 6 μg/ml polybrene, and selected with puromycin. The effect of WWTR1 knockdown was determined by immunoblotting the cell lysates with anti-WWTR1. A luciferase shRNA (shLUC) (The targeting sequence: 5′-C G C T G A G T A C T T C G A A A T G T C-3′) was used as a control.

### Cell lysate preparation and immunoblotting

The cells were rinsed with cold PBS after removal of culture medium and lysed using precooled mammalian cell lysis buffer (40 mM Hepes, ph 7.4, 100 mM NaCl, 1% Triton X-100, 25 mM glycerol phosphate, 1 mM sodium orthovanadate, 1 mM EDTA, 10 μg/ml aprotinin and 10 μg/ml leupeptin) by rocking plates at 4 °C for 30 min. Cell lysates were cleared by centrifugation at 14,000×*g* in a microcentrifuge for 5 min at 4 °C before use.

The SDS-PAGE lysate samples were prepared by addition of 5 × SDS sample buffer directly to the lysates, followed by vortex and denatured at 100 °C for 5 min. After electrophoresis on SDS-PAGE gels, the separated proteins on gels were transferred onto PVDF membranes (Millipore). The membranes were incubated with primary antibodies overnight at 4 °C and with secondary antibodies for 2 h at room temperature. The target proteins were detected by the Western Lightning ECL Detection Kit (Beytime).

### Cell proliferation and migration assays

#### Cell proliferation assay

AGS cells (the shRNA control or the shWWTR1 cells) were cultured in F12 K with 10% FBS at 37 °C plus 5% CO_2_. AGS cells (4 × 10^4^) were seeded in each well of a 12 well culture plate. After cultured at designated time points, the cells were trypsinized and counted under a phase microscope with a hemocytometer. The cell proliferation is evaluated by the cell number increased since seeded. The proliferation assay was repeated at least three times.

#### Cell migration assays

The transwell assay was used in determination of the cell migration. AGS cells were collected and suspended in serum-free F12 K medium. The cells (4 × 10^4^ in 200 μl) were gently added to the upper chamber of Transwell (Corning). F12 K medium with a migration attractant (10% FBS or/and 50 ng/ml EGF) (0.5 ml) was added to the lower chamber. After incubation for a designated time, the cells on the upper side of the separation membrane between the upper and the lower chambers were carefully removed, the cells migrated to bottom side of the membrane were fixed with 4% paraformaldehyde for 30 min and stained with 0.1% crystal violet solution. The stained cells were washed with PBS three times, visualized under a phase microscope and counted under a microscope from three randomly selected fields.

#### Statistical analysis

The association between clinicopathological variables and WWTR1 expression was determined using the Chi square test. Kaplan–Meier survival analysis was conducted from post-surgery to death, stratified by WWTR1 expression status, TNM stage, T stage, N stage, tumor size, and differentiation, respectively. Statistical analysis of clinicopathological and IHC staining data was performed using IBM SPSS software (IBM Corp, Armonk, NY). The difference of the cell migration between cell lines or treatments was analyzed by the Student t-test. *P*-value < 0.05 was considered as statistically significant.
